# Safety Evaluations of Rapamycin Perfluorocarbon Nanoparticles in Ovarian Tumor-Bearing Mice

**DOI:** 10.3390/nano14211752

**Published:** 2024-10-31

**Authors:** Qingyu Zhou, John C. Harding, Ping Fan, Ivan Spasojevic, Attila Kovacs, Antonina Akk, Adam Mitchell, Luke E. Springer, Joseph P. Gaut, Daniel A. Rauch, Samuel A. Wickline, Christine T. N. Pham, Katherine Fuh, Hua Pan

**Affiliations:** 1 Department of Pharmaceutical Sciences, Taneja College of Pharmacy, University of South Florida, Tampa, FL 33612, USA; 2 Molecular Oncology, Oncology Division, Washington University School of Medicine, St. Louis, MO 63110, USA; 3 Medical Oncology, Department of Medicine, Duke University, Durham, NC 27708, USA; 4 Cardiovascular Division, Washington University School of Medicine, St. Louis, MO 63110, USA; 5 Division of Rheumatology, Washington University School of Medicine, St. Louis, MO 63110, USA; 6 Department of Pathology and Immunology, Washington University School of Medicine, St. Louis, MO 63110, USA; 7 John Cochran Veterans Affairs Medical Center, St. Louis, MO 63106, USA; 8 Division of Gynecologic Oncology, University of California, San Francisco, NC 90095, USA; 9 Department of Biomedical Engineering, Washington University in St. Louis, St. Louis, MO 63110, USA

**Keywords:** nanoparticle, safety, pharmacology kinetics, biodistribution, immunity

## Abstract

Nanomedicine holds great potential for revolutionizing medical treatment. Ongoing research and advancements in nanotechnology are continuously expanding the possibilities, promising significant advancements in healthcare. To fully harness the potential of nanotechnology in medical applications, it is crucial to conduct safety evaluations for the nanomedicines that offer effective benefits in the preclinical stage. Our recent efficacy studies indicated that rapamycin perfluorocarbon (PFC) nanoparticles showed promise in mitigating cisplatin-induced acute kidney injury (AKI). As cisplatin is routinely administered to ovarian cancer patients as their first-line chemotherapy, in this study, we focused on evaluating the safety of rapamycin PFC nanoparticles in mice bearing ovarian tumor xenografts. Specifically, this study evaluated the effects of repeat-dose rapamycin PFC nanoparticle treatment on vital organs, the immune system, and tumor growth and assessed pharmacokinetics and biodistribution. Our results indicated that rapamycin PFC nanoparticle treatment did not cause any detectable adverse effects on cardiac, renal, or hepatic functions or on splenocyte populations, but it reduced the splenocyte secretion of IL-10, TNFα, and IL12p70 upon IgM stimulation. The pharmacokinetics and biodistribution results revealed a significant enhancement in the delivery of rapamycin to tumors by rapamycin PFC nanoparticles, which, in turn, led to a significant reduction in ovarian tumor growth. Therefore, rapamycin PFC nanoparticles have the potential to be clinically beneficial in cisplatin-treated ovarian cancer patients.

## 1. Introduction

Cisplatin has shown significant efficacy in the treatment of advanced ovarian cancer after surgical debulking and recurrent ovarian cancer, both as a single agent and in combination with other anticancer drugs [1,2,3]. It has been reported that approximately 40–50% of patients with advanced ovarian cancer developed acute kidney injury after receiving cisplatin during cytoreductive surgery and hyperthermic intraperitoneal chemotherapy [4,5]. Although efforts to protect kidneys from cisplatin-induced toxicity were initiated in 1970s [6], the main clinical option is still supportive measures, such as hydration and/or magnesium replacement [7,8,9,10,11,12,13,14,15,16,17]. Amifostine is used as a nephroprotective rescue agent; however, its own side effects and drug–drug interaction remain problematic, and its interference with anti-cancer treatments requires further evaluation [18,19]. Recently, we have demonstrated that rapamycin PFC nanoparticles protected renal function from cisplatin treatment at an early stage by enhancing autophagy and inhibiting inflammation [20], as well as the safety of the rapamycin PFC nanoparticles in normal mice [21]. However, the safety of its use in tumor-bearing conditions remains to be investigated.

Although phase I clinical trials typically involve the participation of health volunteers, in the oncology field, it is common to incorporate a small cohort of cancer patients in the phase I trials. Therefore, in this study, we focused on understanding pharmacokinetics and biodistribution of rapamycin PFC nanoparticles as well as their effects on vital organs, immune cells, and tumor growth in mice bearing ovarian tumors. Our results indicated that rapamycin PFC nanoparticles did not adversely affect vital organs or alter splenocyte composition, except affecting B cell secretion of IL-10, TNFα, and IL12p70 upon IgM stimulation. A significantly higher amount of rapamycin was detected in the tumors of mice treated with the rapamycin PFC nanoparticles compared to those administrated free rapamycin. Moreover, the treatment with rapamycin PFC nanoparticles significantly inhibited tumor growth, potentially due to the inhibition of angiogenesis.

## 2. Materials and Methods

### 2.1. Nanoparticle Formulation and Characterization

The rapamycin perfluorocarbon nanoparticles were formulated by using the method as previously described [20]. Briefly, the lipid/rapamycin mixture comprises 0.4 mol% rapamycin (Cat No. J62473, Alfa Aesar via FisherSci, Tampa, FL, USA), 1 mol% dipalmitoyl-phosphatidylethanolamine, and 98.6 mol% egg lecithin (Avanti Polar Lipids, Piscataway, NJ, USA). Rhodamine-labelled nanoparticles have 0.4 mol% of 16:0 Liss Rhodamine PE lecithin (Avanti Polar Lipids, Piscataway, NJ, USA) in the lipid mixture. The mixture was dissolved in the solvent, a blend of chloroform and methanol (3:1, *v*/*v*). The solvent was removed under reduced pressure to generate a lipid film using a rotavapor. The lipid film was subsequently dried overnight in a vacuum oven. Following this, the lipid film (2.0%, *w*/*v*) was combined with perfluorooctyl bromide (20%, *w*/*v*) (Gateway Specialty Chemicals, St. Peters, MO, USA) and sterile filtered MilliQ water. The mixture underwent sonication and emulsification at 20,000 psi for 6 passes in an ice bath using an LV-1 Microfluidics emulsifier (Microfluidics, Newton, MA, USA).

The characterization of the rapamycin perfluorocarbon nanoparticles included particle size, poly-dispersity index, and surface charge. Dynamic light scattering was utilized to evaluate particle size and poly-dispersity index, while the nanoparticle surface charge was determined by zeta potential measured by a PALS Zeta Potential Analyzer (Brookhaven Instruments Corp., Holtsville, NY, USA).

For electron microscope (EM) visualization, 25% glutaraldehyde was added to each sample suspension, to a final concentration of 1% glutaraldehyde. The grid was sequentially placed on a drop of sample suspension, dH_2_O, dH_2_O, and 1% aqueous Uranyl Acetate for 10 min, 30 s, 30 s, and 1 min, respectively. Excess liquid was wicked off from the grid with a piece of Whatman paper. The grids were allowed to air dry in a grid box at least 30 min prior to EM imaging.

### 2.2. Cell Culture

Human ovarian tumor cells, OVCAR8 engineered with stable GFP overexpression (CSC-RR0468, www.creative-biogene.com (accessed on 16 November 2021)), were maintained in a humidified atmosphere of 95% air and 5% CO_2_ at 37 °C in DMEM (Cat#: 11965092, Thermo Fisher Scientific, Waltham, MA, USA) with 10% heat inactivated FBS (Cat#: 16140071, Thermo Fisher Scientific).

### 2.3. Nanoparticle Cellular Uptake

Human ovarian tumor cells, OVCAR8 engineered with stable GFP overexpression (CSC-RR0468, www.creative-biogene.com (accessed on 16 November 2021)), were seeded at 100,000 cells in a Delta T Dish 0.17 mm (clear) (Cat#: 04200417C-50, Bioptechs Inc., Butler, PA, USA). Twenty-four hours post seeding, cells were treated with Rhodamine-labelled PFC nanoparticles 1:100 (*v*:*v*) in a complete cell culture medium. After 24 h of nanoparticle treatment, cells were washed three times in PBS with Ca^2+^ and Mg^2+^ (Cat#: 14040133, Thermo Fisher Scientific), fixed with 4% paraformaldehyde in PBS (Cat#: J19943-K2, Thermo Scientific) at room temperature for 10 min, and then washed three times in PBS with Ca^2+^ and Mg^2+^ (Cat#: 14040133, Thermo Fisher Scientific), before confocal imaging. 

### 2.4. Animal Model

Female athymic nude mice (Crl:NU(NCr)-Foxn1nu, 7 weeks old) were purchased from Charles River Laboratories Inc. (Chicago, IL). Human ovarian tumor cell, OVCAR8, engineered with stable GFP overexpression (CSC-RR0468, www.creative-biogene.com (accessed on 16 November 2021)) (1 × 10^6^) suspended in 0.2 mL PBS without Ca^2+^ and Mg^2+^ (Cat #: 10010023, Gibco, via Fisher Scientific, Tampa, FL, USA) was injected subcutaneously into the right flank of the athymic nude mice. Tumor-bearing mice were randomly divided into rapamycin nanoparticle treatment and saline groups (N = 7 for each). Two weeks after tumor inoculation, mice were either treated with rapamycin nanoparticle at 1 ml/kg, twice a week for three weeks, or saline as a control. All animal-related procedures were approved by the Washington University School of Medicine’s IACUC.

### 2.5. Echocardiogram

A mouse echocardiogram was acquired by the Mouse Cardiovascular Phenotyping Core facility at Washington University School of Medicine by using the VisualSonics 2100 Echocardiography System (VisualSonics Inc., Toronto, ON, Canada). The sedation was introduced with avertin (0.005 mL/g). Both two-D and M-mode images were acquired in the long axis and short axis views. Measurements were performed on 3 independently acquired images per animal by investigators who were blinded to the experimental group.

### 2.6. Tissue Preservation and Processing

Seventy-two hours after the last dose, the mice were euthanized for whole blood, heart, liver, kidney, spleen, and tumor collection. The whole blood was centrifuged for obtaining serum for blood chemistry at Washington University School of Medicine Research Animal Diagnostic Laboratory. The tumors were divided in half for either cyro-preservation in O.C.T (Fisher Scientific, Waltham, MA, USA) or 10% formalin. The hearts, livers, and kidneys were preserved in 10% formalin (Sigma, St. Louis, MO, USA). All the tissues in 10% formalin remained in the solution for over 24 h but less than 48 h before being processed into paraffin blocks, sectioned, and stained for H&E at The Anatomic and Molecular Pathology Core Labs at Washington University School of Medicine.

### 2.7. Splenocyte Isolation, Subpopulation Analysis, and Stimulation Evaluation

The spleens were aseptically collected, minced in cold PBS, and then passed through nylon mesh filters to obtain single-cell suspensions. Erythrocytes were lysed by the addition of Tris-NH_4_Cl, washed twice with cold PBS, and counted with trypan blue. The viability of the splenocytes was greater than 95%. The total number of splenocytes per spleen was ~24 × 10^6^. FITC anti-mouse IgD Antibody (Cat#: 405704, BioLegend, San Diego, CA, USA); PE Rat Anti-Mouse CD19 Antibody (Cat#: 553786, BD Biosciences, Franklin Lakes, NJ, USA); PerCP/Cyanine5.5 anti-mouse IgM Antibody (Cat#: 408612, BioLegend); FITC Rat Anti-Mouse Ly-6C Antibody (Cat#: 553104, BD Biosciences); PE anti-mouse CD 69 Antibody (Cat#: 104508, BioLegend); PerCP anti-mouse Ly-6G Antibody (Cat#: 127654, BioLegend); and APC Rat Anti-Mouse CD45R/B220 (553092, BD Biosciences) were used for identification of splenocyte subpopulation. Briefly, 1 × 10^6^ total splenocytes from each spleen were collected and blocked in anti-FcR monoclonal antibody 2.4 G2, before being stained by incubation with the indicated antibodies above at 4 °C for 20 min. Cells were then washed and resuspended for flow-cytometry data collection by a FACSCalibur cell analyzer. The data analysis was performed by using Cell-Quest Pro software (Version 5.1, BD Biosciences). By FACS, we obtained the percentage of each population per sample and calculated an absolute number for all subpopulations. For stimulation evaluation, splenocytes were plated into 96-well U bottom plates (353077, Falcon), at 5 × 10^5^ cells per well in K10 media. Cell samples were stimulated in triplicates by anti-IgM (Cat#: 115-005-020, Jackson ImmunoResearch Laboratories) at a final concentration of 20 µg/mL, anti-IgM (115-005-020, Jackson ImmunoResearch Laboratories), or LPS (Cat#: L4391, Sigma) at a final concentration of 40 µg/mL. After 3 days in a cell culture incubator, splenocytes were collected by spin down in the U bottom 96-well plates at 1500 RPM for 5 min. Supernatants were used for BD^TM^ Cytometric Bead Array and the Infiltratory Kit (Cat#: 552364, BD Biosciences) for cytokine production evaluations. Cells were stained with CD45R/B220-APC (553092, BD Biosciences), and proliferation was measured by using a FACSCalibur cell analyzer.

### 2.8. Single-Dose Pharmacokinetic Study

Female athymic nude mice (Hsd: Athymic Nude-Foxn1nu; 5–6 weeks old) were purchased from Envigo (Indianapolis, IN, USA). Rapamycin used for intravenous (IV) administration was dissolved in Tween 80/polyethylene glycol (50:50, *v*/*v*) to prepare as the stock solution at a concentration of 1 mg/mL. The stock solution was diluted with 0.9% NaCl saline to obtain a solution with the final rapamycin concentration of 0.1 mg/mL prior to use. Rapamycin nanoparticles used for IV administration contained 0.1 mg/mL of rapamycin. OVCAR8 human ovarian tumor cells (5 × 10^6^) suspended in 0.2 mL Matrigel (Catalog number: 354234. Corning Inc., Corning, NY, USA) were injected subcutaneously into the right flank of the athymic nude mice. Tumor-bearing mice were randomly divided into unformulated rapamycin and rapamycin nanoparticle groups (N = 6 for each). Two weeks after tumor inoculation, each animal had its left carotid artery catheterized for IV dosing and blood sampling prior to the IV administration of unformulated rapamycin or rapamycin nanoparticle. Each animal was then given a single IV bolus injection of 30 μL of 0.1 mg/mL of rapamycin solution or rapamycin nanoparticle suspension through the left carotid artery, and the catheter was flushed with 100 μL of saline. Whole blood samples were collected at 0 min (pre-dose); 5, 20, and 40 min; and 1, 2, 4, 6, 8, and 20 h post dose. An aliquot of 10 μL of the whole blood sample was immediately mixed with 0.4 μL of 0.5 M potassium EDTA and stored at −80 °C before drug analysis. Following the blood sampling at 20 hr post dose, all animals were euthanized under isoflurane anesthesia and perfused to remove blood. The heart, lung, liver, kidney, spleen, stomach, small intestine, brain, bladder, ovary, and tumor were collected at autopsy and stored at −80 °C before being subjected to LC-MS/MS analysis of rapamycin concentrations in tissues. All animal-related procedures were approved by the University of South Florida’s IACUC.

### 2.9. LC-MS/MS Assay of Rapamycin in Blood and Tissues

#### 2.9.1. Sample Processing

Samples were thawed and homogenized with two parts water (*w*/*vol*) and two 2.5 mm zirconia/silica beads (Biospec Products Inc., Bartlesville, OK, USA) in a blunted 0.5 mL polypropylene (PP) conical vial by vigorous agitation in a Fast-Prep apparatus (Thermo-Savant, Thermo Fisher Scientific) at speed 4 for 40 s at room temperature. Next, 100 µL aliquot of the obtained tissue homogenate, 10 µL of 200 ng/mL desmethoxyrapamycin, DMR (Internal standard. Supelco, Inc. Bellefonte, PA, USA), and 300 µL of chloroform were added into a 1.5-mL PP conical vial and vigorously agitated in the Fast-Prep apparatus (speed 4, 40 s). After discarding the (upper) aqueous phase, 200 µL of the organic phase was transferred to a 12 × 75 mm glass tube and evaporated to dryness by a gentle stream of nitrogen. The residue was then reconstituted with a 50 µL precipitate mix (70% methanol, 30% 0.3 M ZnSO_4_ in water) and transferred into an LC/MS/MS injection vial. In the case of the blood, 10 µL of blood, 10 µL of 200 ng/mL DMR, and 40 µL of precipitate mix (70% methanol, 30% 0.3 M ZnSO_4_ in water) were added into 0.2-mL PCR tube and vigorously agitated in the Fast-Prep apparatus (speed 4, 40 s). After centrifugation at 13,600× *g* for 5 min at room temperature, 40 µL of supernatant was transferred into an injection vial for analysis.

#### 2.9.2. Liquid Chromatography-Tandem Mass Spectrometry (LC-MS/MS)

The LC-MS/MS analysis of rapamycin was performed on a Shimadzu 20 A series LC system interfaced with an Applied Biosystems/SCIEX API 4000 QTrap MS/MS spectrometer (AB Sciex LLC, Framingham, MA, USA). Analyst software (version 1.6.1) was used for mass parameters tuning, data acquisition, and quantification. LC conditions were (1) column: Phenomenex Kinetex (C18, 3 × 4 mm, #AJ0-4287. Phenomenex. Torrance, CA, USA); (2) mobile phase A: 10 mM ammonium acetate, 0.1% acetic acid, 3% methanol, in DI water; (3) mobile phase B: 10 mM ammonium acetate, 0.1% acetic acid, 3% DI water, in methanol; (4) flow rate: 0.5 mL/min; (5) elution gradient: 0–1 min, 50% B; 1.0–1.5 min, 50–100% B; 1.5–2.0 min, 100% B, 2.0–2.2 min, 100–50% B; and (6) run time: 3.5 min. A diverter valve was used to send the flow to MS/MS only between 0.6 and 2 min. The autosampler was at 4 °C; injection volume was 20 µL. The compounds were individually infused as 100 ng/mL solutions in 50%A/50%B at 10 µL/min flow rate and ionization/ion path parameters were optimized. Parent/daughter quantifier (qualifier) ions utilized were rapamycin (931.5/864.5(882.4)) and DMR (901.5/852.5(834.5)).

#### 2.9.3. Calibration and Quantification

Calibration samples (*n* = 6) were prepared by adding pure standard (rapamycin, Supelco) of the measured compound to tissue homogenate in the appropriate range. The following are ranges used (the lower value representing also the LLOQ at 80% accuracy limit, all other calibrator levels at 85% accuracy limit): 2.4–1000 ng/mL (blood) and 0.7–90 ng/mL (tissue). Signal area integration, calibration, and quantification were performed within Analyst v 1.6.1 software. The response of the peak area standard/internal standard to nominal concentration was linear with r = 0.999 or better.

### 2.10. Pharmacokinetic Analysis

A two-compartment open model with first-order elimination from the central compartment ($C=Ae^{-\alpha t}+Be^{-\beta t}$) was used to characterize rapamycin disposition in blood. Values of the volume of the central compartment (V_C_), the volume of distribution at steady state (V_ss_), the volume of distribution of the slow disposition phase (V_β_), total clearance (CL), and half-lives for the fast (t_1/2,α_) and slow (t_1/2,β_) disposition phases were calculated as described previously [21].

### 2.11. Immunofluorescence Staining and Imaging

Immunofluorescence staining was conducted on tumors embedded in O.C.T and on paraffin-embedded hearts, livers, and kidneys. Cryo-sectioning of O.C.T-embedded tumors was performed to obtain 8 µm sections, which were then fixed in 4% PFA before staining. Paraffin-embedded tissues were sectioned at 5 µm using a microtome prior to staining. Heat-induced antigen retrieval was applied for the paraffin-embedded tissues.

The CD31 primary antibody (Abcam, Waltham, Boston, MA, USA) was incubated with the sections overnight at 4 °C, followed by incubation with secondary antibodies labeled with Alex 594 (Abcam, Waltham, Boston, MA, USA) at room temperature for 30 min. Slides were mounted with the VECTASHIELD antifade mounting medium with DAPI (Vector Laboratories, Newark, CA, USA) before imaging. The imaging process utilized an Olympus dark-field microscope equipped with a HAMAMATSU digital camera C11440 at 20× or 40× magnification.

### 2.12. Statistics

Unless otherwise indicated, results were expressed as mean ± standard error of mean (SEM). Statistical analyses of pharmacokinetic data were performed using Graph Pad Prism 8.0 (Graph Pad, San Diego, CA, USA). Comparison of means between two independent groups was made using the two-sample *t* test. The statistical significance of differences was attributed at *p* < 0.05.

## 3. Results

### 3.1. Rapamycin PFC Nanoparticles Characterization and Cellular Uptake

**Figure 1:**
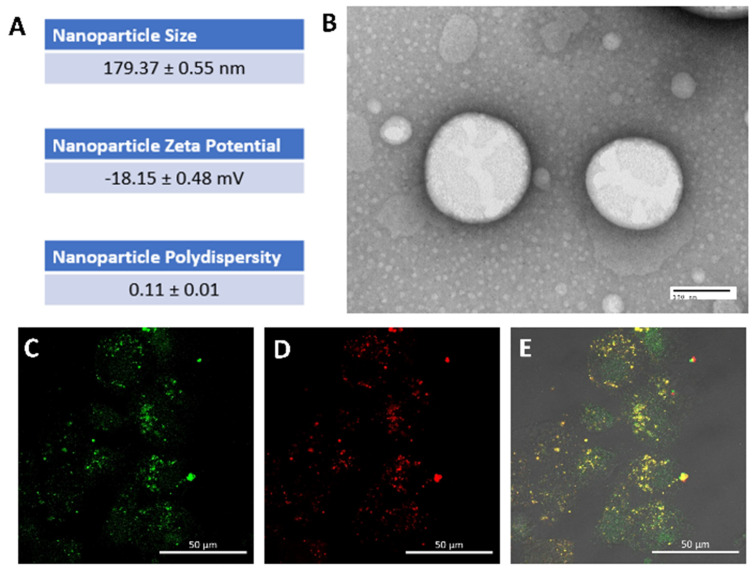
Physical characteristics of rapamycin PFC nanoparticles and their cellular uptake by OVCAR8-GFP cells. (**A**). Particle size, zeta potential, and polydispersity were evaluated by dynamic light scattering measurements (Mean ± SEM). (**B**). Representative electron microscope image of rapamycin nanoparticles (scale bar: 100 nm). (**C**,**D**). Representative confocal images indicate Rhodamine-PFC nanoparticles ((**D**); red) cellular uptake by OVCAR8-GFP cells (**C**; green) 24 h after nanoparticle treatment. (**E**). Merged image from (**C**,**D**). (scale bar: 50 µm).

Perfluorocarbon (PFC) nanoparticles consist of a perfluorocarbon core enveloped by a lipid monolayer. The lipid monolayer is customizable and can serve as a carrier for various functional entities, including therapeutic agents. The physical attributes of these PFC nanoparticles include particle size, surface charge, and polydispersity index. Using the dynamic light scattering technique, the measured nanoparticle size was found to be 179.37 ± 0.55 nm, with a polydispersity index of 0.11 ± 0.01. The polydispersity index of the PFC nanoparticles characterizes a given sample’s size distribution, offering insights into their size heterogeneity. A polydispersity index of 0.11 ± 0.01 indicated a narrow size distribution in the formulation. Besides particle size and size distribution, the surface charge of the nanoparticles was characterized by zeta potential, −18.15 ± 0.48 mV, which provided electrostatic repels among the nanoparticles. The LC-MS-MS quantification indicated that the concentration of rapamycin in nanoparticles was 102.23 ± 2.47 µg/mL. To visualize the uptake of PFC nanoparticles by ovarian cancer cells, OVCAR8-GPF cells were utilized. The distinct red fluorescent signals (Figure 1D) emanated from the green OVCAR8-GPF cells (Figure 1C), indicating that Rhodamine-labelled PFC nanoparticles were taken up by the OVCAR8-GPF cells within 24 h of treatment.

### 3.2. Effects of Rapamycin PFC Nanoparticles Treatment on Vital Organs

#### 3.2.1. Cardiac Function

**Figure 2:**
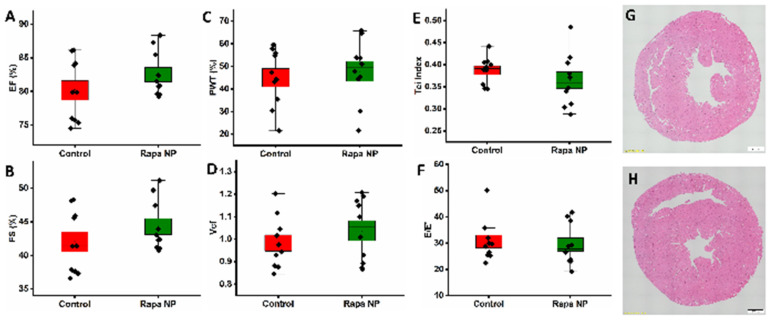
Cardiac function and morphology of mice with (Rapa NP) or without (Control) rapamycin PFC nanoparticles treatment. (**A**) Ejection fraction (EF), (**B**) fractional shortening (FS), (**C**) posterior wall thickening (PWT), (**D**) velocity of circumferential fiber shortening (Vcf), (**E**) Tei Index, (**F**) E/E’ (Mean ± SEM). (**G**,**H**) Representative H&E staining of the cross-section of the heart from control (**G**) and rapamycin PFC nanoparticle-treated groups (**H**), respectively. Magnification: 10× and scale bar: 500 µm.

An echocardiogram was performed to evaluate left ventricular systolic and diastolic function after repeated doses of rapamycin PFC nanoparticles. To assess left ventricular global systolic function, left ventricular ejection fraction (EF), left ventricular fractional shortening (FS), left ventricular posterior wall thickening (PWT), and velocity of circumferential fiber shortening (Vcf) were evaluated. The EF of the control and the rapamycin PFC nanoparticles-treated group was 80.16 ± 1.46% and 82.49 ± 1.06%, respectively (Figure 2A) (*p* = 0.11). The FS of the control and the rapamycin PFC nanoparticles-treated group was 42.01 ± 1.47% and 44.28 ± 1.20%, respectively (Figure 2B) (*p* = 0.12). The PWT of the control and the rapamycin PFC nanoparticles-treated group was 45.02 ± 4.02% and 47.81 ± 4.34%, respectively (Figure 2C) (*p* = 0.32). To assess left ventricular systolic function that is pre-load independent, the Vcf was measured. The Vcf of the control and the rapamycin PFC nanoparticles-treated group was 0.98 ± 0.04 and 1.04 ± 0.04, respectively (Figure 2D) (*p* = 0.17). To evaluate left ventricular diastolic function, Tei Index and E’/E were calculated. The Tei Index, also known as myocardial performance index, of the control and the rapamycin PFC nanoparticles-treated group was 0.39 ± 0.01 and 0.36 ± 0.02, respectively (Figure 2E) (*p* = 0.15). The E’/E of the control and the rapamycin PFC nanoparticles-treated group was 30.61 ± 2.48 and 29.46 ± 2.52, respectively (Figure 2F) (*p* = 0.37). These echocardiogram measurements indicated that the rapamycin PFC nanoparticle treatment did not alter left ventricular function. The H&E staining of the cross-section of hearts from the control (Figure 2G) and the rapamycin PFC nanoparticles-treated (Figure 2H) groups illustrated the normal tissue structure and morphology.

#### 3.2.2. Hepatic Function

**Figure 3:**
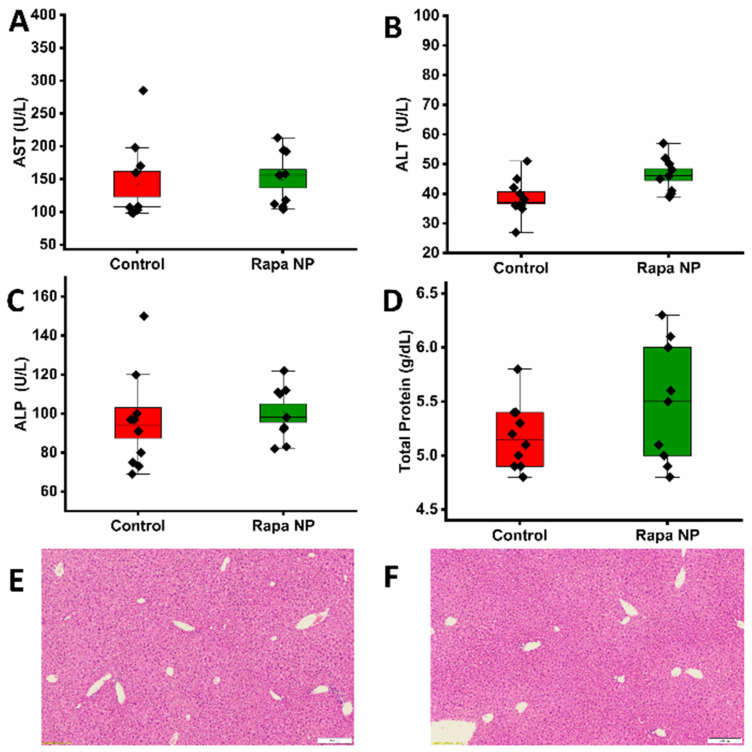
Hepatic function and morphology of mice with (Rapa NP) or without (Control) rapamycin PFC nanoparticles treatment. Hepatic function was assessed by liver enzymes. (**A**) Aspartate aminotransferase (AST), (**B**) alanine transaminase (ALT), and (**C**) alkaline phosphatase (ALP), as well as (**D**) total protein levels (Mean ± SEM). (**E**,**F**) Representative H&E staining of liver from control (**E**) and rapamycin PFC nanoparticle treated group (**F**), respectively. Magnification: 20×, scale bar: 200 µm.

To assess the hepatic function, a blood test was performed to evaluate liver enzymes, aspartate aminotransferase (AST), alanine transaminase (ALT), and alkaline phosphatase (ALP), as well as total protein levels. The AST of the control and the rapamycin PFC nanoparticles-treated group was 142.70 ± 20.03 and 150.56 ± 14.00 U/L, respectively (Figure 3A). The ALT of the control and the rapamycin PFC nanoparticles-treated group was 38.60 ± 1.68 and 46.44 ± 1.99 U/L, respectively (Figure 3B). The ALP of the control and the rapamycin PFC nanoparticles-treated group was 95.20 ± 7.93 and 100.33 ± 4.68 U/L, respectively (Figure 3C). Total protein levels, including albumin and globulins, of the control and the rapamycin PFC nanoparticles-treated group were 5.18 ± 0.10 and 5.48 ± 0.19 g/dL, respectively (Figure 3D). The liver enzymes and total protein levels of the mice in both groups were all within normal ranges. Consistently, the liver H&E staining results from the control (Figure 3E) and the rapamycin PFC nanoparticles-treated (Figure 3F) groups illustrated the normal tissue structure and morphology.

#### 3.2.3. Renal Function

**Figure 4:**
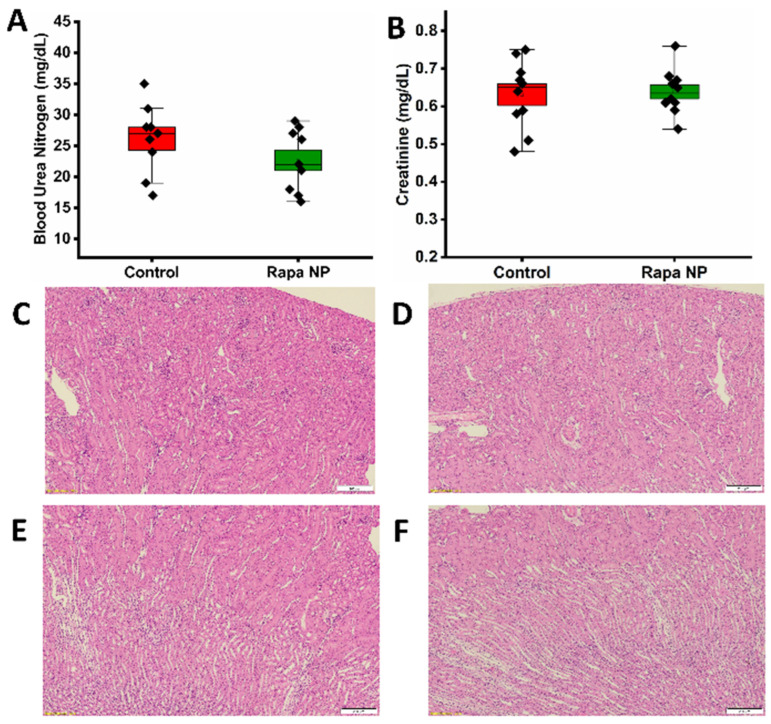
Renal function and morphology of mice with (Rapa NP) or without (Control) rapamycin PFC nanoparticles treatment. Renal function was assessed by (**A**) Blood Urea Nitrogen and (**B**) Creatinine (Mean ± SEM). (**C**,**D**) Representative H&E staining of renal cortex from control (**C**) and rapamycin PFC nanoparticle-treated group (**D**), respectively. E-F. Representative H&E staining of renal medulla from control (**E**) and rapamycin PFC nanoparticle-treated group (**F**), respectively. Magnification: 20×, scale bar: 200 µm.

To evaluate renal function, a blood test was performed to assess blood urea nitrogen (BUN) and creatinine. The BUN of the control and the rapamycin PFC nanoparticles-treated group was 26.11 ± 1.86 and 22.67 ± 1.67 mg/dL, respectively (Figure 4A). The creatinine of the control and the rapamycin PFC nanoparticles-treated group was 0.63 ± 0.03 and 0.64 ± 0.02 mg/dL, respectively (Figure 4B). Both the BUN and creatinine of both groups were within the normal ranges. Consistently, the H&E of kidney cortex and medulla from the control (Figure 4C,E, respectively) and the rapamycin PFC nanoparticles-treated (Figure 4D,F, respectively) groups suggested normal tissue structure and morphology.

### 3.3. Effects of Rapamycin PFC Nanoparticles Treatment on Splenocytes

Given rapamycin’s immunosuppressive properties, the effects of rapamycin PFC nanoparticles on splenocytes were investigated regarding the number of total splenocytes, the subpopulations of splenocytes, and the proliferation and cytokine productions of splenocytes under stimulations.

#### 3.3.1. The Number of Total and Subpopulation of Splenocytes as Well as Splenocyte Proliferation Under Different Stimulations

**Figure 5:**
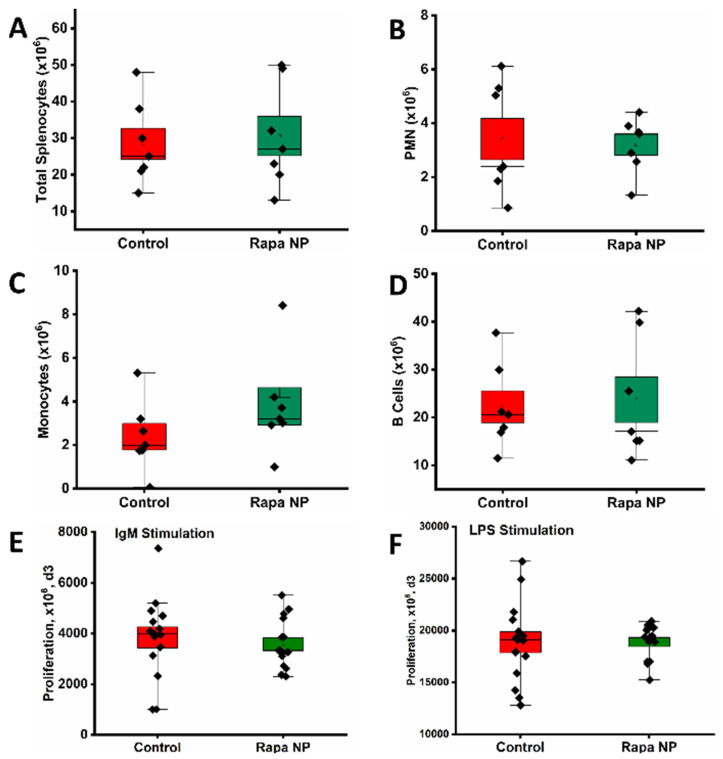
Effects of rapamycin PFC nanoparticle treatment on splenocytes. (**A**) Total number of splenocytes, (**B**–**D**) composition of the splenocytes and the number of subpopulation of splenocytes, (**B**) polymorphonuclear neutrophils (PMNs), (**C**) monocytes, and (**D**). (**B**) Cells were not significantly altered by rapamycin PFC nanoparticle treatment. (**E**,**F**) Splenocyte proliferation evaluated on day 3 (d3) post IgM (**E**) and LPS (**F**) stimulation, respectively, was not affected by rapamycin PFC nanoparticle treatment (Mean ± SEM). Reported results are from of mice with (Rapa NP) or without (Control) rapamycin PFC nanoparticles treatment.

The total splenocytes in the spleens of the control and the rapamycin PFC nanoparticles treatment groups were 28.43 × 10^6^ ± 4.27 × 10^6^ and 30.57 × 10^6^ ± 5.37 × 10^6^, respectively (Figure 5A) (*p* = 0.76). Polymorphonuclear neutrophils (PMNs), monocytes, and B cells were identified in both the control and the rapamycin PFC nanoparticles-treated group. The total numbers of PMNs in the control and the rapamycin PFC nanoparticles treatment groups were 3.41 × 10^6^ ± 0.77 × 10^6^ and 3.2 × 10^6^ ± 0.39 × 10^6^, respectively (Figure 5B) (*p* = 0.81). The total numbers of monocytes in the control and the rapamycin PFC nanoparticles treatment groups were 2.38 × 10^6^ ± 0.61 × 10^6^ and 3.78 × 10^6^ ± 0.86 × 10^6^, respectively (Figure 5C) (*p* = 0.21). The total numbers of B cells in the control and the rapamycin PFC nanoparticles treatment groups were 22.24 × 10^6^ ± 3.32 × 10^6^ and 23.70 × 10^6^ ± 4.76 × 10^6^, respectively (Figure 5D) (*p* = 0.81). The proliferation of the splenocytes was assessed under the stimulation of either IgM or LPS. Three days after the stimulation of IgM, the total numbers of cells in the control and the rapamycin PFC nanoparticles treatment groups were 3842.80 × 10^6^ ± 411.97 × 10^6^ and 3586.68 × 10^6^ ± 254.67 × 10^6^, respectively (Figure 5E) (*p* = 0.60). Three days after the stimulation of LPS, the total numbers of cells in the control and the rapamycin PFC nanoparticles treatment groups were 18,869.20 × 10^6^ ± 1001.67 × 10^6^ and 18,876.12 × 10^6^ ± 421.16 × 10^6^, respectively (Figure 5F) (*p* = 0.99). These results indicated that the treatment didn’t alter the total number of splenocytes, the composition of splenocyte subpopulation, or the splenocyte proliferation upon IgM and LPS stimulations.

#### 3.3.2. The Splenocyte Cytokine Productions Under Different Stimulations

**Figure 6:**
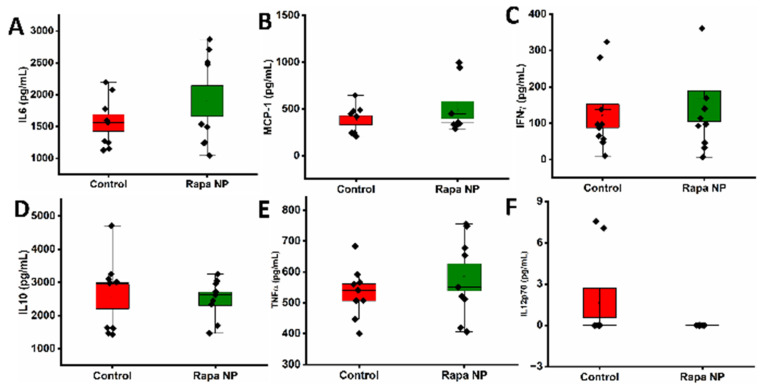
Effects of rapamycin PFC nanoparticle treatment on splenocyte activity in response to LPS stimulation. LPS induced IL6 (**A**), MCP-1 (**B**), IFNγ (**C**), IL10 (**D**), TNFα (**E**), and IL12p70 (**F**) productions by splenocytes were not affected by rapamycin PFC nanoparticle treatment (Mean ± SEM). Reported results are from of mice with (Rapa NP) or without (Control) rapamycin PFC nanoparticles treatment.

The splenocytes’ productions of cytokines, IL6, MCP-1, IFNγ, IL10, TNF-α, and IL12p70 under the stimulation of LPS and IgM were investigated. Under the LPS stimulation, the splenocytes’ cytokine productions were not significantly different between the control and the rapamycin PFC nanoparticles treatment groups. IL6 productions in the control and the rapamycin PFC nanoparticles treatment groups were 1557.08 ± 132.32 pg/mL and 1903.14 ± 241.18 pg/mL, respectively, (Figure 6A) (*p* = 0.23). MCP-1 productions in the control and the rapamycin PFC nanoparticles treatment groups were 378.42 ± 50.76 pg/mL and 488.31 ± 92.05 pg/mL, respectively, (Figure 6B) (*p* = 0.31). IFNγ productions in the control and the rapamycin PFC nanoparticles treatment groups were 102.39 ± 32.38 pg/mL and 135.30 ± 42.78 pg/mL, respectively, (Figure 6C) (*p* = 0.62). IL10 productions in the control and the rapamycin PFC nanoparticles treatment groups were 2577.93 ± 372.60 pg/mL and 2504.87 ± 199.72 pg/mL, respectively, (Figure 6D) (*p* = 0.86). TNF-α productions in the control and the rapamycin PFC nanoparticles treatment groups were 533.74 ± 27.49 pg/mL and 582.73 ± 44.01 pg/mL, respectively, (Figure 6E) (*p* = 0.36). The IL12p70 level in the control group was 1.62 ± 1.07 pg/mL, but it was not detectable in the rapamycin PFC nanoparticle group (0 ± 0 pg/mL. *p* = 0.08) (Figure 6F).

**Figure 7:**
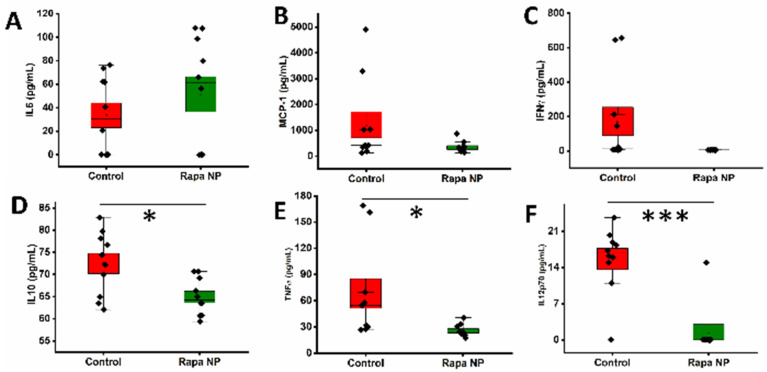
Effects of rapamycin PFC nanoparticle treatment on splenocyte activity in response to IgM stimulation. IgM-induced IL6 (**A**), MCP1 (**B**), and IFNγ (**C**) production by splenocytes was not affected by rapamycin PFC nanoparticle treatment, while IgM-induced IL10 (**D**), TNF-α (**E**), and IL12p70 (**F**) production by splenocytes was significantly reduced in the mice that received rapamycin PFC nanoparticle treatment (Mean ± SEM). (*: *p* < 0.05; ***: *p* < 0.001). Reported results are from of mice with (Rapa NP) or without (Control) rapamycin PFC nanoparticles treatment.

Under the IgM stimulation, the splenocytes’ cytokine productions of IL6, MCP-1, IFNγ were not significantly different between the control and the rapamycin PFC nanoparticles treatment groups. However, IL10, TNF-α, and IL12p70 production was significantly lower in the rapamycin PFC nanoparticles treatment group, compared to the control group. IL6 production in the control and the rapamycin PFC nanoparticles treatment groups was 33.55 ± 10.42 pg/mL and 51.59 ± 14.97 pg/mL, respectively, (Figure 7A) (*p* = 0.34). MCP-1 production in the control and the rapamycin PFC nanoparticles treatment groups was 1212.17 ± 504.54 pg/mL and 339.40 ± 68.09 pg/mL, respectively, (Figure 7B) (*p* = 0.10). IFNγ production in the control and the rapamycin PFC nanoparticles treatment groups was 171.84 ± 82.81 pg/mL and 5.95 ± 0.14 pg/mL, respectively, (Figure 7C) (*p* = 0.06). Notably, the IL10 production in the rapamycin PFC nanoparticle groups was significantly reduced as compared with the control group (64.95 ± 1.32 pg/mL versus 72.46 ± 2.28 pg/mL. *p* = 0.01) (Figure 7D). Likewise, the TNF-α production in the rapamycin PFC nanoparticle group control was significantly reduced as compared with the control (25.65 ± 2.22 pg/mL versus 53.15 ± 16.81 pg/mL. *p* = 0.02) (Figure 7E), and the IL12p70 production the rapamycin PFC nanoparticles-treated group was significantly reduced as compared with the control (1.50 ± 1.50 pg/mL versus 15.64 ± 2.04 pg/mL. *p* < 0.001) (Figure 7F).

### 3.4. Pharmcokenitics and Biodistribution

**Figure 8:**
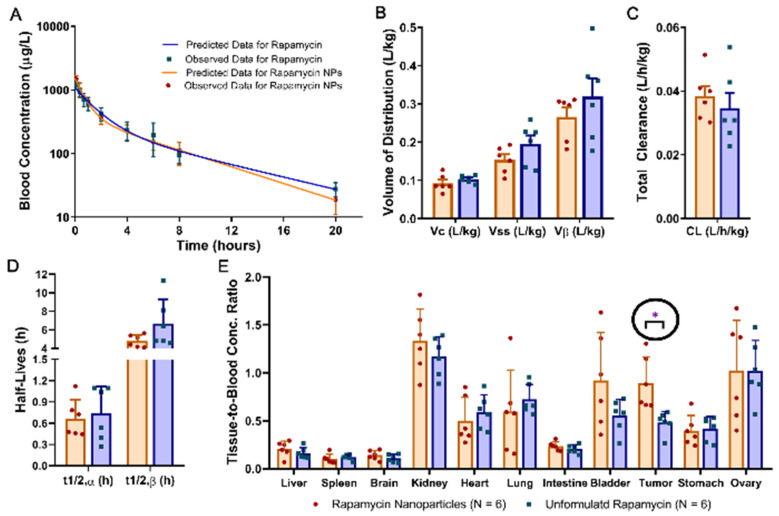
Systemic disposition kinetics and tissue distribution of rapamycin following the intravenous (IV) administration of 30 μL of 0.1 mg/mL of unformulated rapamycin or rapamycin nanoparticles in female OVCAR8 tumor-bearing athymic nude mice. (**A**). A bi-exponential decline in rapamycin blood concentrations was observed in the rapamycin blood concentration–time profile after the IV dosing of either unformulated rapamycin or rapamycin nanoparticles (N = 6 for each group) to the tumor-bearing athymic nude mice, suggesting that a two-compartment open model with first-order elimination from the central compartment can be used to describe the disposition kinetics of rapamycin. (**B**). Comparison of the volume of the central compartment (Vc), volume of distribution at steady state (Vss), and volume of distribution of the slow disposition phase (V_β_) values between the rapamycin nanoparticle and unformulated rapamycin groups (N = 6 for each) showed no statistically significant differences (*p* > 0.05 for all). (**C**). Comparison of the total clearance (CL) values between the rapamycin nanoparticle and unformulated rapamycin groups (N = 6 for each) showed no statistically significant differences (*p* > 0.05) (**D**). Comparison of the half-life values for the fast (t_1/2_,α) and slow (t_1/2_,β) disposition phase between the rapamycin nanoparticle and unformulated rapamycin groups (N = 6 for each) showed no statistically significant differences (*p* > 0.05). (**E**). Comparison of the ratios of tissue-to-blood concentrations obtained at 20 h after IV dosing of the unformulated rapamycin or rapamycin nanoparticles. The mean tumor-to-blood concentration ratio in the rapamycin nanoparticle groups was significantly higher than in the unformulated rapamycin group (*p* < 0.05). No significant differences in the mean rapamycin tissue-to-blood concentration ratios in heart, lung, liver, kidney, spleen, stomach, small intestine, brain, bladder, and ovary were found between unformulated rapamycin and rapamycin nanoparticle groups. Data are expressed as mean ± standard deviation (SD). SD is denoted by the error bars. * *p* < 0.05 as compared with the unformulated rapamycin group using the independent sample *t* test.

The systemic disposition kinetics of rapamycin in OCVAR8-eGFP tumor-bearing athymic nude mice following a single IV bolus injection of unformulated rapamycin or rapamycin nanoparticles was best described by the two-compartmental open model with first-order elimination from the central compartment (Figure 8A). Results of the PK analysis showed that the mean V_C_, V_ss_, V_β_, t_1/2_,α, and t_1/2_,β values in the rapamycin nanoparticle group were decreased by 10%, 21%, 17%, 9% and 28%, respectively, as compared with those in the unformulated rapamycin group, whereas the mean C_L_ value in the rapamycin nanoparticle group was 11% greater than in the unformulated rapamycin group (Figure 8B–D. Table 1). However, the results of independent sample *t*-test indicated that the differences in V_C_, V_ss_, V_β_, C_L_, t_1/2_,α, and t_1/2_,β values were not statistically significant between the unformulated rapamycin and rapamycin nanoparticle groups (Figure 8B–D). The rapamycin biodistribution in various tissues was assessed by use of the ratios of tissue-to-blood rapamycin concentration determined at 20 h after the IV bolus injection of unformulated rapamycin and rapamycin nanoparticles. As shown in Figure 6E, the tissue-to-blood concentration ratios in liver, brain, kidney, intestine, bladder, spleen, heart, lung, stomach and ovary in the rapamycin nanoparticle group were similar to those in the unformulated rapamycin group. Results of the independent sample *t*-test indicated that the tumor-to-blood concentration ratio in the rapamycin nanoparticle group was significantly higher than in the unformulated rapamycin group (*p* < 0.05), whereas the differences in the tissue-to-blood concentration ratios of other tissues were not statistically significant between the two study groups (Figure 8E). There results suggested similar disposition kinetics but different tumor distribution of unformulated rapamycin and rapamycin-loaded nanoparticles. Formulating rapamycin into PFC nanoparticles facilitated the delivery of rapamycin into tumors.

### 3.5. Rapamycin PFC Nanoparticles Treatment Inhibited Ovarian Tumor Growth

**Figure 9:**
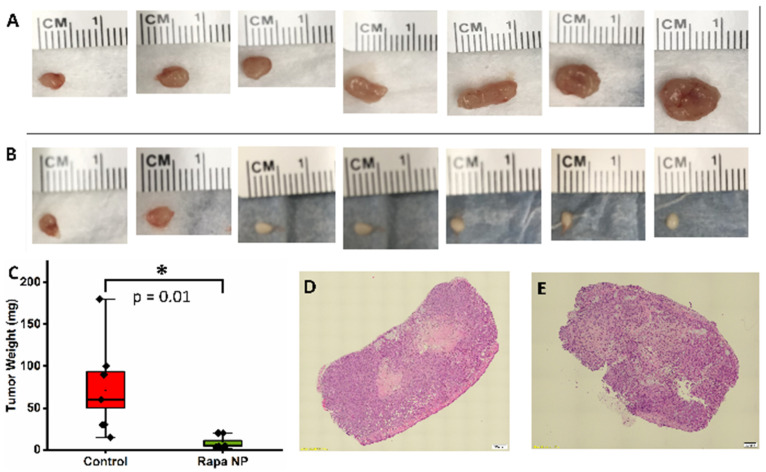
Rapamycin PFC nanoparticle treatment significantly inhibited tumor growth. (**A**). Photos of the tumors from control group. (**B**). Photos of the tumors from rapamycin PFC nanoparticle-treated mice. (**C**). Quantification of tumor weights from control or rapamycin PFC nanoparticle-treated (Rapa NP) mice. (**D**). Representative H&E staining of the tumor from control group (Magnification: 10×; scale bar: 500 µm). (**E**). Representative H&E staining of the tumor from rapamycin PFC nanoparticle-treated group (Magnification: 10×; scale bar: 200 µm). (*: *p* < 0.05).

As shown in Figure 9A,B, the tumors from the mice in the control group were larger than those from the mice in the rapamycin PFC nanoparticles treatment group. The tumor weights from the control mice and the rapamycin PFC nanoparticles-treated mice were 72 ± 22 mg and 8 ± 3 mg (Figure 9C). The treatment significantly reduced tumor growth (*p* = 0.01). The H&E staining of the tumors from the control and the rapamycin PFC nanoparticles-treated mice are shown in Figure 9D,E, respectively.

### 3.6. Rapamycin PFC Nanoparticles Treatment Inhibited Tumoral Vessel Establishement Without Impairing Blood Vessels in the Vital Organs

**Figure 10:**
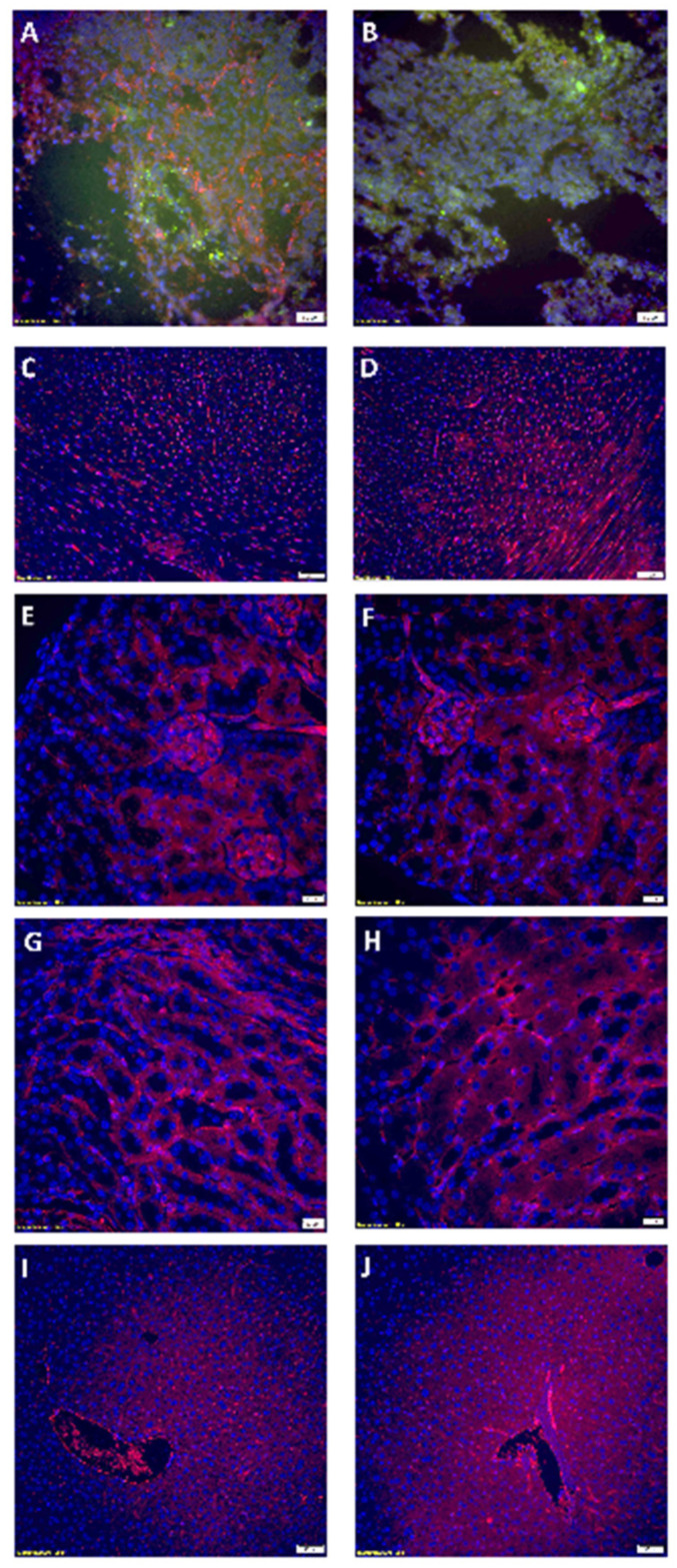
Rapamycin PFC nanoparticles inhibit tumoral angiogenesis without affecting normal vessels, illustrated by CD31 staining (Red). (**A**,**B**). Tumor from control group (**A**) and the rapamycin PFC nanoparticles-treated group (**B**) (magnification: 20×, scale bar: 50 µm) (Green: Human ovarian tumor cell, OVCAR8, engineered with stable GFP overexpression). (**C**,**D**). Heart from control group (**C**) and the rapamycin PFC nanoparticles-treated group (**D**) (magnification: 20×, scale bar: 50 µm). (**E**,**F**). Renal cortex from control group (**E**) and the rapamycin PFC nanoparticles-treated group (**F**) (magnification: 40×, scale bar: 20 µm). (**G**,**H**). Renal medulla from control group (**G**) and the rapamycin PFC nanoparticles-treated group (**H**) (magnification: 40×, scale bar: 20 µm). (**I**,**J**). Liver from control group (**I**) and the rapamycin PFC nanoparticles-treated group (**J**) (magnification: 20×, scale bar: 50 µm). Blue: DAPI staining.

As illustrated in Figure 10A, in the tumor from the control group, OVCAR8 tumor cells engineered with stable GFP overexpression (green fluorescence) were surrounded by a well-established tumoral vessel network, as indicated by CD31 staining (red fluorescence). In contrast, the tumor from the rapamycin PFC nanoparticles-treated group showed a marked diminution of the tumoral vessel network, although some CD31 staining was still observed (Figure 10B). CD31 staining was also performed on the heart (Figure 10C: control and Figure 10D: the rapamycin PFC nanoparticles-treated), the cortex of the kidney (Figure 10E: control and Figure 10F: the rapamycin PFC nanoparticles-treated), the medulla of the kidney (Figure 10G: control and Figure 10H: the rapamycin PFC nanoparticles-treated), and the liver (Figure 10I: control and Figure 10J: the rapamycin PFC nanoparticles-treated). No significant differences in blood vessel presence in these vital organs were observed.

## 4. Discussion

Rapamycin is a macrolide that was isolated originally from Streptomyces hygroscopicus in a soil sample from Easter Island, or Rapa Nui, hence the name rapamycin [22,23,24]. Currently, rapamycin is approved by the FDA as an oral immunosuppressant to prevent transplant rejection by blocking interleukin-2 signaling in B and T cells [25]. Mammalian Target of Rapamycin (mTOR) is a high molecular weight serine/threonine kinase, which is an integral node in protein synthesis and cell growth signaling. mTOR exists in two complexes, mTORC1 and mTORC2. Rapamycin directly inhibits mTORC1 but not mTORC2 [26,27,28,29,30]. Inhibition of mTORC1 also may result in reduction of downstream NF-κB signaling [31,32] and consequently suppress tubular inflammation.

Rapamycin is also known to induce autophagy, thereby inducing a cell survival program that enables useful recycling of amino acids via nonspecific degradation of long-lived proteins and dysfunctional organelles [33]. Indeed, in a previous study we show that rapamycin PFC nanoparticle pretreatment mitigates cisplatin-induced acute kidney injury through simultaneously enhancing autophagy and inhibiting inflammation in a preclinical mouse model [20]. Ovarian cancer patients routinely receive cisplatin chemotherapy but can quickly develop drug resistance due, in part, to cessation to prevent cisplatin-induced nephrotoxicity. Therefore, we focused this study on evaluating the safety of rapamycin PFC nanoparticles in a tumor-bearing model, specifically examining their effects on vital organ function, immune cell function, and tumor growth by utilizing ovarian tumor-bearing mice.

In this study, we utilized echocardiography to assess the effects of the rapamycin PFC nanoparticle treatment on left ventricular systolic and diastolic functions. We evaluated left ventricular ejection fraction (EF), left ventricular fractional shortening (FS), left ventricular posterior wall thickening (PWT), velocity of circumferential fiber shortening (Vcf), Tei Index, and E’/E. EF and FS are commonly used measurements of left ventricular systolic function, with EF providing a percentage of blood ejected per heartbeat and FE reflecting the extent of myocardial shortening. If there are abnormalities in regional wall motion, FS provides a more accurate indication of the left ventricular global function. PWT assesses the thickening of the left ventricular posterior wall, which enables identification of hypertrophy or structural changes. Vcf evaluates the rate of circumferential fiber shortening, providing insights into the contractile performance of the heart. Tei Index indicates global cardiac function, incorporating both the systolic and diastolic phase to assess the overall efficiency of the heart in pumping blood. E’/E ratio assesses diastolic function by comparing velocities of mitral annulus and left ventricular filling. Taking together, these metrics provide a comprehensive view of left ventricular function and structure [34]. Our results indicated that the rapamycin PFC nanoparticle treatment did not affect these key metrics and suggested normal cardiac function. We and others have previously demonstrated that rapamycin treatment is cardiacprotective in aged animals [35,36]. A recent systematic review article suggested that rapamycin has cardiacprotective effects in studies related to human aging, although further investigation is needed [37]. In this study, young adult mice with normal cardiac function were utilized, and results suggested a cardiac safety profile in a younger cancer patient population. On the other hand, according to statistics from the Ovarian Cancer Research Alliance, 24.5% of ovarian cancer cases are diagnosed in woman aged 55 to 63, while 24.1% are diagnosed in women aged 65 and 74. Additionally, half of all ovarian cancer patients are diagnosed after the age of 63. Since 17-month-old mice approximately correlate to 60-year-old humans [38], our follow-up studies will include aged mice to evaluate both safety and therapeutic effects, with the hypothesis that the rapamycin PFC nanoparticle can improve cardiac function of the aged tumor-bearing mice.

It was reported that 12-week-old male mice developed fatty liver after receiving rapamycin at a dose of 80 µg per day for 8 days [39], without showing an increase in markers of liver cirrhosis, which is a potential adverse outcome of fatty liver [40]. This lack of cirrhosis may be attributed to rapamycin-induced inhibition of canonical NF-αB activation [40]. As shown in Figure 3F, our rapamycin PFC nanoparticle treatment did not cause fatty liver, possibly due to the lower rapamycin dose over a longer period of treatment time. To evaluate liver function, liver enzymes, AST, ALT, and ALP were measured. These enzymes provide important information about the liver’s ability to process substances. ALT is considered one of the most specific indicators of liver injury. An elevated level of ALT can suggest liver inflammation or damage. Our results indicated that the ALT levels of the mice in the rapamycin PFC nanoparticle-treated group were within the normal range. AST is found in the liver, heart, and muscles. Elevated AST levels can indicate liver damage but are less specific than ALT, since high levels can result from issues with the heart and/or muscles. However, our results indicated normal ALT levels in the rapamycin PFC nanoparticle-treated animals. ALP is involved in the breakdown of proteins and is present in the liver, bones, and bile ducts. The normal ALP levels in the rapamycin PFC nanoparticle-treated animals indicated that the liver is normal and there is no bile duct obstruction. Total protein levels are measured alongside liver enzymes. The normal total protein levels in the rapamycin PFC nanoparticle-treated animals suggested that the liver is functioning properly in terms of protein synthesis. It is worth noting that the absolute values of AST, ALT, and ALP from female athymic nude mice reported here are different from those of male C57BL/6 mice [21], although the values are all within the normal range. These results suggest potential gender and genetic background differences.

Consistent with our previous preclinical study [21], rapamycin PFC nanoparticle treatment did not impair renal function, as shown in Figure 4, indicated by normal BUN and creatinine levels as well as normal kidney morphology. Major urinary proteins are present in mouse urine, such that wild-type C57BL/6NCr mice have a urinary protein concentration of 73.7 ± 8.2 mg/dL [41]. Therefore, we didn’t assess urine protein concentrations after obtaining normal BUN and creatinine levels in both groups. However, it would be beneficial to evaluate the effect of the rapamycin PFC nanoparticle treatment on urinary protein concentration by using Mup-KO mice, which have a urinary protein concentration of 17.9 ± 1.8 mg/dL [41]. On the other hand, a recent meta-analysis of clinical trials found that treatment with rapamycin derivatives significantly increases the relative risk of all-grade acute kidney injury (AKI) but not high-grade AKI. However, the treatment does not significantly affect the incidence rates of either all-grade or high-grade AKI in cancer patients [42]. Although this increased relative risk may be related to the fact that most of the clinical trials included in the meta-analysis focused on renal cell carcinoma [42], these findings should be taken into consideration for potential clinical development of the rapamycin PFC nanoparticles.

The potent immunosuppressive effect of rapamycin is well recognized. As shown in Figure 6, the results indicated that treatment with the rapamycin PFC nanoparticle did not affect the total number of splenocytes, their composition, or splenocyte proliferation in responding to stimuli. Since the immunodeficient athymic nude mice lack T cells, we evaluated cytokine expressions by PMNs, monocytes, and B cells in response to LPS and IgM stimulations. Based on the results shown in Figure 6, the rapamycin PFC nanoparticle treatment did not alter those immune cell responses to the presence of LPS. On the other hand, the splenocytes produced a significantly lower amount of IL-10, TNF-α, and IL12p70 in response to IgM stimulation after the repeat-dose treatment with rapamycin PFC nanoparticles, which was likely to be attributable to the immunosuppressive effect of rapamycin.

In this study, the blood concentration–time profile of rapamycin obtained from the OCVAR8-eGFP tumor-bearing female athymic nude mice that were given a single IV dose of unformulated rapamycin or rapamycin nanoparticles demonstrated a biexponential behavior of rapamycin disposition. IV administration of rapamycin nanoparticles resulted in slight decreases in the mean volume distribution values (Figure 8B) and half-lives of the fast and slow disposition phase (Figure 8D) and a slight increase in the total clearance value (Figure 8C) as compared with the unformulated rapamycin. This observation can be attributed to the reduced binding affinity of rapamycin nanoparticles for red blood cells and tissues, which is in part due to the reduced lipophilicity of the nanoparticles as compared with the unformulated rapamycin. The reduced binding affinity of rapamycin nanoparticles for red blood cells also leads to the increased elimination of rapamycin in the blood circulation and a quicker transport of rapamycin from blood to tissue, which were manifested by the increased total clearance and decreased half-life of the fast disposition phase (t_1/2_,α), respectively, as compared with the unformulated rapamycin (Figure 8C,D). The increased elimination of rapamycin in the blood circulation following the IV administration of rapamycin nanoparticles was also reflected by the decreased half-life of the slow disposition phase (t_1/2_,β) as compared with the administration of unformulated rapamycin (Figure 8D).

Overall, there is not a significant difference observed regarding the pharmacokinetics of rapamycin administrated either unformulated or formulated into nanoparticles. However, the pharmacokinetics measured by rapamycin were consistent with those measured by its carrier, PFC nanoparticles, as reported previously [35], indicating the stability of the rapamycin incorporation in the PFC nanoparticles. Different from normal vasculature, leaky blood vessels in tumors provide enhanced permeability and retention (EPR), which is associated with disproportionate accumulation of PFC nanoparticles in the tumor. The lipid membrane of PFC nanoparticles provide the “stick and stay” adhesion to the cell membrane in the tumor, allowing the sustained release of rapamycin molecules from the PFC nanoparticles [43]. In particular, the increase in the tumor-to-blood concentration ratios was statistically significant (*p* < 0.05. Figure 8E). This result suggests that rapamycin nanoparticles improve the uptake of rapamycin in tumors, compared to unformulated rapamycin. It is worth noting that the PK and biodistribution differ between female athymic nude mice reported here and male C57BL/6 mice [21]. These observations indicate the importance of including both male and female participants, as well as candidates from different genetic backgrounds, in the recruitment for clinical trials.

It is crucial that treatments aimed at reducing cancer treatment-induced toxicity do not promote tumor growth. Therefore, we assessed the effects of the rapamycin PFC nanoparticles on tumor growth using tumor-bearing mice. As demonstrated in Figure 9, the rapamycin PFC nanoparticle inhibited tumor growth. It has been recognized that rapamycin inhibits tumor growth through anti-angiogenetic mechanisms [44]. Consistently, our results illustrated that rapamycin PFC nanoparticle treatment inhibited tumoral vessel development comparing to control (Figure 10A,B). We are currently evaluating the molecular mechanisms underlying the anti-cancer effects of rapamycin PFC nanoparticles.

## 5. Conclusions

Our results demonstrated that PFC nanoparticles improved the tumor uptake of rapamycin following the systemic administration that served to inhibit tumor growth. The overall findings suggest that rapamycin PFC nanoparticle treatment is generally safe and poses low risk to vital organ functions. Further evaluation is warranted to elucidate the dose–effect relationship for the treatment suppression of IL10, TNF-α, and IL12p70 in monocytes, PMNs, and B cells under IgM stimulation as potential infections during the treatment for cancer patients.

## Figures and Tables

**Table 1:** Systemic disposition kinetics of rapamycin in mice after single IV bolus injection of 30 μL of 0.1 mg/mL of unformulated rapamycin and rapamycin nanoparticle.

	Unformulated Rapamycin (N = 6)	Rapamycin Nanoparticles (N = 6)
AUC (μg·h/L)	3877 ± 1131	3419 ± 639
t_1/2,α_ (h)	0.737 ± 0.382	0.668 ± 0.266
t_1/2,β_ (h)	6.67 ± 2.67	4.81 ± 0.67
V_C_ (L/kg)	0.103 ± 0.010	0.092 ± 0.022
V_ss_ (L/kg)	0.195 ± 0.054	0.153 ± 0.034
V_β_ (L/kg)	0.319 ± 0.117	0.266 ± 0.059
CL (L/h/kg)	0.035 ± 0.012	0.038 ± 0.008

Note: Data were presented as mean ± standard deviation (SD).

## Data Availability

The data presented in this study is available in the article.
